# The INHERIT Model: A Tool to Jointly Improve Health, Environmental Sustainability and Health Equity through Behavior and Lifestyle Change

**DOI:** 10.3390/ijerph15071435

**Published:** 2018-07-07

**Authors:** Nina van der Vliet, Brigit Staatsen, Hanneke Kruize, George Morris, Caroline Costongs, Ruth Bell, Sibila Marques, Timothy Taylor, Sonia Quiroga, Pablo Martinez Juarez, Vojtech Máca, Milan Ščasný, Iva Zvěřinová, Fimka Tozija, Dragan Gjorgjev, Geir Arild Espnes, Jantine Schuit

**Affiliations:** 1 National Institute for Public Health and the Environment (RIVM), Centre for Sustainability, Environment and Health, 3720 BA Bilthoven, The Netherlands; brigit.staatsen@rivm.nl (B.S.); hanneke.kruize@rivm.nl (H.K.); 2 Tilburg School of Social and Behavioral Sciences, University of Tilburg, 5000 Tilburg, The Netherlands; a.j.schuit@uvt.nl; 3 European Centre for Environment and Human Health, University of Exeter Medical School, Truro TR1 3HD, UK; geomorris55@hotmail.co.uk (G.M.); timothy.j.taylor@exeter.ac.uk (T.T.); 4 EuroHealthNet, 1040 Brussels, Belgium; c.costongs@eurohealthnet.eu; 5 Institute of Health Equity, UCL, London WC1E 7HB, UK; r.bell@ucl.ac.uk; 6 Instituto Universitário de Lisboa (ISCTE-IUL), CIS-IUL, 1649-026 Lisboa, Portugal; sibila.marques@iscte-iul.pt; 7 Department of Economics, Universidad de Alcalá, 28801 Alcalá, Spain; sonia.quiroga@uah.es (S.Q.); pablo.martinezj@uah.es (P.M.J.); 8 Charles University, Environment Centre (CUNI), 162 00 Prague, Czech Republic; vojtech.maca@czp.cuni.cz (V.M.); milan.scasny@czp.cuni.cz (M.Š.); iva.zverinova@czp.cuni.cz (I.Z.); 9 The Institute of Public Health of the Republic of Macedonia (IJZRM), 1000 Skopje, Macedonia; ftozija@t.mk (F.T.); dgjorgjev@gmail.com (D.G.); 10 NTNU Center for Health Promotion Research, Norwegian University of Science and Technology, 7030 Trondheim, Norway; geir.arild.espnes@ntnu.no

**Keywords:** integrated models, environmental health, behavior, behavioral change, equality, sustainability, food

## Abstract

The need for analysis and action across the interrelated domains of human behaviors and lifestyles, environmental sustainability, health and inequality is increasingly apparent. Currently, these areas are often not considered in conjunction when developing policies or interventions, introducing the potential for suboptimal or conflicting outcomes. The INHERIT model has been developed within the EU-funded project INHERIT as a tool to guide thinking and intersectoral action towards changing the behaviors and lifestyles that play such an important role in today’s multidisciplinary challenges. The model integrates ecological public health and behavioral change models, emphasizing inequalities and those parts of the causal process that are influenced by human behaviors and lifestyles. The model was developed through web-based and live discussions with experts and policy stakeholders. To test the model’s usability, the model was applied to aspects of food consumption. This paper shows that the INHERIT model can serve as a tool to identify opportunities for change in important −food-related behaviors and lifestyles and to examine how they impact on health, health inequalities, and the environment in Europe and beyond. The INHERIT model helps clarify these interrelated domains, creating new opportunities to improve environmental health and health inequality, while taking our planetary boundaries into consideration.

## 1. Introduction

This paper describes the development of a conceptual model to analyze and support action on the interrelated fields of environmental sustainability, health and equity, with the aim to achieving a triple win. The need for analysis and action across these fields is increasingly apparent. Human activity in the Anthropocene is critically affecting natural systems with serious consequences for health and wellbeing [1,2,3]. Many behaviors, lifestyles and the forces that shape them are inherently unhealthy and damaging to the environment. For example, recent patterns of industrialization and urbanization, while conferring great benefits for humanity, are associated with changes in levels of physical activity and diets, and they put pressures on green spaces [4,5]. Western diets now contain high levels of animal protein, sugar and fats, contributing to obesity and related diseases. In addition, meat and dairy products account for approximately 25% of the environmental impacts from consumption of products in the EU, and meat production is one of the leading causes of biodiversity loss [6,7]. 

Anthropogenic changes to environments in a certain location can have health-relevant impacts on populations living in that location, and these impacts can be direct (e.g., inhalation of pesticides used in agriculture) or indirect by influencing health-related behaviors (e.g., an obesogenic environment that may encourage an unhealthy diet and sedentary lifestyle) [8]. In addition, current anthropogenic activities can affect populations elsewhere and future generations through their impact on natural systems. For example, our transport and consumption patterns generate local pollutants, but also greenhouse gasses that contribute to global climate change, increasing (amongst others things) the occurrence of extreme weather events that have detrimental impacts on ecosystems and communities throughout the world [9,10]. Some of these impacts are already visible, whereas others will only become apparent after years or even decades [3,11].

Last but not least, environmental exposure is often socially patterned, with more disadvantaged populations being more frequently exposed to environmental risks. This results in health inequalities [12,13]. For example, across all countries there are clear inequalities in mortality and health status between socioeconomic groups, influenced by environmental factors, but also by social and psychological factors [14,15]. In addition, more disadvantaged populations are more likely to be affected by changes related to climate change, which will widen health inequalities even further in the future [16].

Today’s behaviors and lifestyles, which lie at the root of many 21st century challenges, are hugely complex to understand and even more complex to change. Behavior exists within a complex system that creates and sustains both behavior itself and the environments that shape it. By implication, the same policy or intervention will seldom be equally as effective for all individuals or in all population groups, potentially widening health inequalities [17]. Therefore, a thorough analysis of behavior and its determinants, including how these differ between population groups, is a prerequisite when designing or choosing effective interventions and policies [18]. The health sector has been studying the role of behavior and lifestyles in health for decades [19] and there is a growing body of literature on pro-environmental behavior (consciously performing actions that reduce the impact of one’s activities on the environment or even enhance the quality of the environment) [20,21]. A promising development is research on simultaneously changing health and sustainability behaviors [22]. An important realization from the field of environmental psychology is that physical environments and individuals influence each other, which makes understanding the interactions and the forces that govern them especially important [21,23]. 

Although the issues set out above cross disciplinary boundaries and require intersectoral collaboration at a policy and intervention level, health and environmental sectors often still work in silos [24]. The different perspectives held by stakeholders from diverse disciplines, should ideally be transformed to a shared understanding of the challenge [4]. Promising ways to stimulate this shared understanding include the Health in All Policies (HIAP) approach, which encourages considering health in policy making across sectors that influence health, and the more recent Environment in all Policies (EiAP) approach, which integrates a consideration of environmental issues into policy makers’ agendas. In addition, the Whole of Society approach goes further, acknowledging the contribution and roles of all relevant stakeholders, from families to intergovernmental organizations, and from communities to the private sector [25,26,27]. However, working together with other sectors has proved to be a challenging task, due to differences in culture, training, aims, structures and jargon. Working together initially takes time, building trust and active networking [28]. An innovative approach is necessary that addresses social, economic, political and scientific complexities [29]. Conceptual models may help researchers, policymakers and decision makers gain a better understanding of the complex issues involved. Models help in various ways, especially by engaging multidisciplinary stakeholders, facilitating dialogue and helping to overcome disciplinary and policy boundaries [30,31,32]. Models can also be used to find or develop actions that are effective and acceptable to those involved, and help to find ways to more accurately evaluate these actions. Predictably, issues like climate change and health inequality are difficult to frame using simple linear representations. They are, after all, invariably products of many dynamic and disparate influences in complex interaction. Models that aim to address the complexity of such challenges must walk a fine line between retaining enough simplicity to be usable in practice (a characteristic usually found in linear representations), while taking sufficient account of the complexity that can so easily render simple policy solutions ineffective.

The unique challenges now confronting human society have generated many models and integrated conceptual frameworks. For example, the coupled natural and human systems framework (CHANS), which are integrated frameworks showing the complex systems in which humans and nature interact. CHANS are widely used in ecology and sustainability studies and have been shown to be valuable in linking different disciplines and promoting the identification of new relationships that might otherwise be overlooked [33]. The (environmental, ecological) public health community has generated other tools, such as the tested and widely applied Drivers-Pressures-State-Exposure-Effects (DPSEEA) model and its derivatives [8,34,35], the framework for integrated environmental health impact assessment of systemic risks [36], and the European Commission’s analytical framework for mapping and assessment of ecosystems and their services, linking socioeconomic systems with ecosystems [37]. In addition, a multidisciplinary conceptual framework of environmental quality and quality of life was developed in an attempt to advance the field of urban development, environmental quality and human well-being [23]. Others have recently argued for the importance of an integrating framework for achieving the Sustainable Development Goals, encouraging the development of integrating frameworks to support a more coordinated approach for operationalizing the SDGs [38].

**Figure 1:**
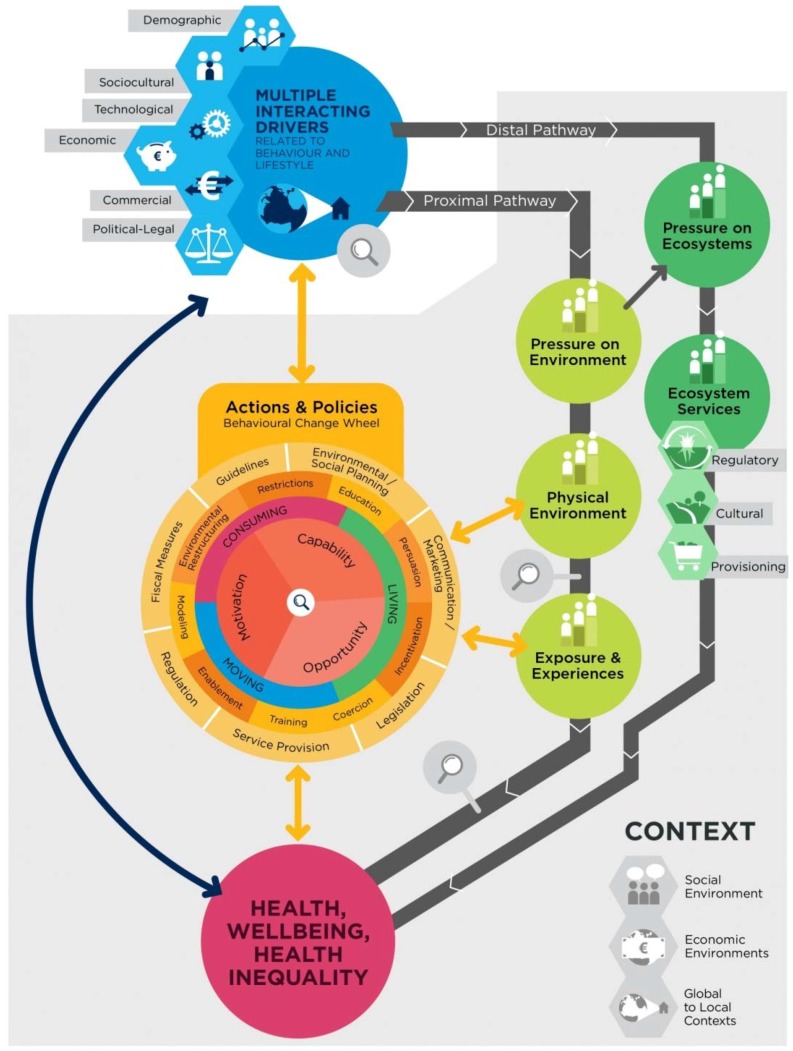
The INHERIT model (the INHERIT model integrates the ecologically enriched DPSEAA model (right side of the model) and Behavioural Change Wheel (left side of the model) bringing together ecological public health and behavior. In addition, icons with individuals standing on different levels throughout the pathways represent inequalities. Magnifying glasses represent spots in the model where behavior plays an important role, either interacting with drivers or modifying effects from the physical environment, exposures to this environment and health, wellbeing and health inequalities impacts.).

The model presented in this paper is a product of, and foundation for, the 4-year EU-funded Intersectoral Health and Environment Research for InnovaTion (INHERIT) project. INHERIT is a research project that aims to understand how lifestyles and behaviors can be changed in order to achieve a triple win of promoting health, environmental sustainability and equality, in the areas of living, moving and consuming. This paper presents the INHERIT model (see Figure 1), and demonstrates how the model can serve as a tool or framework to help understand the interrelationships between the fields of health, sustainability and equality, emphasizing the role of behavior. The paper also aims to demonstrate how the model can help to identify and evaluate (intersectoral) actions aimed at behaviors and lifestyles to achieve a so-called triple win of promoting health, environmental sustainability and equity, contributing to the SDGs.

## 2. Materials and Methods

### 2.1. Procedure

The INHERIT model was developed in an iterative process, with the collaborative efforts of an interdisciplinary team comprising of, among others, scientists and professionals from the environmental and public health domains, psychologists, economists and sociologists. The model was developed through multiple discussion sessions and bilateral discussions in the form of meetings and online interactions, using existing models as a basis. This led to continued revisions and gradual development of the model. Finally, two facilitated sessions were held with a group of (policy) stakeholders to discuss usability of the model. The model was created in parallel with and is informed by a review of current status and opportunities for positive change across Europe towards a triple win of health, sustainability and equity [39]. The review highlights the complex interrelationships that exist around health, sustainability and equity challenges and opportunities, and the undeniable contribution of human behaviors and lifestyles. A key message from the review is that for policies that aim to achieve the triple win to be effective, they need to be grounded in theory and take into account the complexity of interactions with human behaviors. To test the model’s usability, the model was applied to the case of food consumption. The examples used were informed in part by the INHERIT review [39] and in part by other recent literature.

#### 2.1.1. Integrating the DPSEEA Model with a Behavioral Model

The INHERIT model integrates an ecological public health model with a behavioral change model, and emphasizes inequalities and those parts of the causal process that are influenced by human behavior and lifestyles. The ecological public health model is called the Driver-Pressure-State-Exposure-Effect-Action model (DPSEEA) [40,41], which was expanded for use in a national policy context, creating the modified DPSEEA model [8]. In 2015, a further iteration was proposed: the ecosystems-enriched DPSEEA [34]. DPSEEA is reasonably well established in environmental health circles [42,43]. See Table 1 for the key contributions of these models.

In the eDPSEEA model, behavior was presented simply as one element of the context, alongside other mediators of the relationships between environment, exposure and health impacts. For the purpose of INHERIT, this still underplayed the essential role that behaviors and lifestyles play in securing the triple win of health, sustainability and equality, and hence, an explicit incorporation of behavior was deemed necessary. Therefore, the INHERIT model retains the key components of the eDPSEEA model, but the model was augmented by (1) including a behavioral dimension and (2) including an inequality dimension, thereby aiming to secure a more sophisticated and policy relevant framework for analyzing and influencing behavior and inequalities in pursuit of the triple win. 

The goal of incorporating behavior more explicitly is not only to emphasize that many policies tackling the nexus of health, inequality and environmental sustainability involve behavior change, but also to emphasize the need for a more in-depth analysis of behavior when analyzing problems and shaping policy in this complex area. In the field of environmental psychology, the theory of planned behavior [44,45] and the norm-activation model [46] are commonly used for pro-environmental behavior. In the INHERIT model, an action-focused behavioral model, the Behavioural Change Wheel (BCW), which is applicable to a broad range of behaviors (both health and environmental) was incorporated. Michie et al. [47] have developed the BCW as a systematic tool to aid intervention design and theory development. The BCW has already been described extensively elsewhere and will be briefly explained in the results section of this paper. In addition, magnifying glass icons are added at four points in the INHERIT model (see Figure 1), to represent behavioral hotspots, where it is believed behavior and lifestyles can be influenced most effectively by policies and actions within EU countries in order to secure a healthier, more equal and sustainable world. 

An additional feature in the INHERIT model is the expansion of the contextual bubble (visualized by a shaded background in Figure 1) to encapsulate both pathways below the multiple interacting drivers. This recognizes the reality that each transition between the components in both pathways (proximal and distal) is influenced by interacting external factors and is likely to be more complex than implied by the linear configuration depicted. A consequence of this is that, at each stage in the transition from drivers to impacts on health and wellbeing, there is potential to generate inequalities. This has been made explicit by adding an inequality component throughout both pathways) and by including health inequalities as an impact of both pathways. 

#### 2.1.2. Expert and Stakeholder Consultations

Two interactive sessions took place in which the utility of the INHERIT model was explored. The first was held in The Netherlands in 2017 and attendees were INHERIT partners who were not involved in the development of the model together with external experts in the area of transport, consumption, environment and health promotion (n = 19). Central to the first session was the question of whether the model was suitable for the evaluation of interventions aimed at changing behaviors and lifestyles planned for the next stages of the INHERIT project. The second session was a stakeholder consultation, and was held in Belgium shortly after the Dutch session. It brought together representatives and experts from various European, national and academic institutions such as the European Commission, EUREGHA, WHO, University of Exeter and Belgium Agency of Health Care (n = 21). The session began with an explanation of the model, followed by a plenary group discussion on the suitability and usability of the model for the work of the attendees and as a tool to help analyze and apply it to different challenges of health and sustainability. 

## 3. Results

First, the INHERIT model will be briefly introduced and described, followed by a more detailed discussion of the model. Although the model is intended to be applied to a wide range of fields such as active transport, energy efficient housing or green space, the model will be illustrated using examples from the field of food consumption. Finally, the results from testing the model in the stakeholder sessions are presented.

### 3.1. Description of the INHERIT Model

The INHERIT model (see Figure 1) comprises of several building blocks. On the right side, the model distinguishes two pathways: the proximal and distal pathways. Both pathways are initiated by multiple interacting drivers operating at the level of society that generate pressures that change the physical environment, or in the case of the distal pathway, disrupt so-called ecosystem services. By including both pathways in the model, policy− and decision makers are encouraged to think about the impacts of current actions not only on the “here and now”, but also on the “there and then” [48]. The changes to physical environments and ecosystem services result in modified exposures and experiences of the population. Icons showing individuals standing at different levels throughout both pathways represent the fact that drivers create unequal distributions of pressures and resulting physical environments, at both group and individual level. The grey area surrounding the pathways represents the influence of different contextual factors and behaviors that subsequently modify whether and to what extent certain exposures lead to certain effects on health and wellbeing, resulting in health inequalities.

In addition, two behavioral components can be identified in the model. The first one is on the left side of the model, represented by the Behavioural Change Wheel (BCW). The BCW consists of a behavioral system surrounded by two rings of intervention functions and policy categories [49]. The BCW offers a way to analyze relevant behaviors and identify ways to change these behaviors and the environments that influence them. The second behavioral component is the addition of behavioral hotspots, in the form of magnifying glass icons at three points in the model. These are points in the model where behavior plays a significant role, making those points important entry points for action. 

In the next section, the different building blocks of the INHERIT model will be discussed in more detail, using the case of food consumption and production to demonstrate application of the model. These building blocks consist of: (1) the proximal pathway, (2) the distal pathway, (3) a feedback loop, (4) inequalities and context, (5) behavior and (6) interventions and policy action. 

### 3.2. Explanation of the INHERIT Model

#### 3.2.1. The Proximal Pathway

The proximal pathway represents the traditional environmental health perspective [50], in which certain changes to an environment near in time and space to an affected population can result in health and wellbeing impacts for individuals within that population. Multiple interacting drivers initiate the pathway, impacting either positively or negatively and can take many forms, including demographic, economic or technologic drivers. The drivers are related to and interact with behaviors and lifestyles. This makes the drivers one of the behavioral hotspots (indicated by a magnifying glass), and an important entry point for action as both pathways and their impacts start here.

These driving forces together produce pressures that change or sustain a particular physical environment. Sometimes, only a single driver may cause a certain pressure, but in most cases, pressures are the product of a complex and dynamic set of interacting drivers. These pressures vary from the release of harmful substances to the development of certain infrastructure or facilities. Importantly, drivers can also lead to beneficial pressures on environments (e.g., technologies that lead to reduced released of harmful substances, or political drivers that lead to increased availability of healthy food stores). 

The lower part of the pathway, from physical environment to health, wellbeing and health inequalities, is about an individual’s exposure to certain environments, leading to health and wellbeing impacts. This exposure-impact mechanism can be chemical or microbiological, but also (socio-) psychological [8]. Nowadays, health is often defined as “the ability to adapt and self-manage in the face of social, physical, and emotional challenges” [51]. This ‘positive health’ concept places a stronger focus on health and wellbeing than on health problems or disease. 

#### 3.2.2. Food Example of the Proximal Pathway

When applying the model to the case of food consumption, the continuing growth of the global population, is a (demographic) driver of current food consumption and production, as it results in a growing number of mouths to feed [52]. The economic driver of increased income levels has allowed greater accessibility to food, and it is associated with increased meat and fat consumption [53]. A related technological driver is increased agricultural intensification, as it has increased agricultural productivity and the availability of food. At the same time, a positive technological driver is the development of innovative technologies that allow the production of plant-based protein sources (such as seaweed or beans). Trade policies and market liberalization have led to the globalization of food markets, supporting the increasing availability of a wide variety of foods throughout the year [54]. As current lifestyles demand quick, easy meals, this stimulates the production of highly processed foods and establishment of take-away shops is stimulated. For more examples of behavioral drivers, see Section 3.2.8 on the behavioral hotspots in the INHERIT model. The mix of drivers including agricultural intensification and food market globalization leads to pressures such as land clearing to grow crops, reduced fertility of soil, use of pesticides and fertilizers. These can lead to local agricultural land with depleted soil and reduced biodiversity, or to agricultural areas containing a high concentration of pesticides in crops, air or water [55]. Other types of pressures include the development of fast food outlets, and the high availability and affordability of highly processed foods and meat and dairy products in supermarkets [56]. In contrast, the development of plant-based protein source technologies may lead to a physical environment with higher availability of healthier, more sustainable products [57].

Applying the lower part of the pathway to the case of food consumption, an example of an exposure would be living in an area with a relatively high accessibility to fast food, which is positively associated with obesity [58]. Western populations are exposed to an environment filled with processed foods, and have shifted from minimally processed diets to diets containing pre-processed foods high in energy, fat and sugar, promoting obesity. In addition, our high level of (red and processed) meat consumption has been found to increase risks of cancer and premature death [59,60,61]. Behavior plays a mediating role between physical environments, exposures and experiences to these environments, and subsequent health and wellbeing impacts, which will be described in Section 3.2.8.

#### 3.2.3. Distal Pathway

The distal pathway on the right side of the model is about the “there and then”, and encourages thinking about the environmental sustainability of our current activities. The drivers as described in the proximal pathway can also have health-relevant environmental impacts in other parts of the world, or have impacts that may only become apparent after years or even decades [48,50]. The drivers create pressures on ecosystems, either directly or through pressures on environments in the proximal pathway. Ecosystem services are the natural benefits that humans gain from their natural environment and healthy functioning ecosystems. Ecosystems offer several types of services, from cultural (e.g., recreational, spiritual) to regulatory (e.g., control of climate, soil quality) and provisioning (e.g., food and water production) [62]. 

For health impacts to occur, people have to experience or be exposed to these ecosystem service disruptions. However, the INHERIT model does not explicitly visualize exposures and experiences in the distal pathway, because the focus of the INHERIT project lies mainly on current behaviors and exposures to environments. Human health and wellbeing may be affected by damaged ecosystem services in many ways, for example through reduced availability of material goods obtained from the environment (e.g., food, fiber and fuel); reduced security and direct damage to mental and physical health [62]. The distal pathway also ends with impacts on health inequalities, because disadvantaged populations are often hit hardest by negative impacts of damaged ecosystem services. It has been predicted that adverse health effects of climate change will be much more likely in low-income countries and among vulnerable population groups [16,63]. These populations have fewer resources or basic material needs to cope with natural disasters, food− or housing insecurities. 

#### 3.2.4. Food Example of the Distal Pathway

The same drivers that were mentioned in the example of the proximal pathway example also affect ecosystems and populations in the distal pathway. Our global food system accounts for 30% of all anthropogenic greenhouse gas emissions, thereby contributing to global warming [64]. Our meat-centric meals generate about nine times more greenhouse gas emissions than equivalent plant-based meals [65]. Twenty-four percent of the 11.5 billion hectares of vegetated land globally has undergone soil degradation due to human activity, severely affecting the provisioning services that land can offer in terms of food [55]. Moreover, provisioning services may be threatened when rainforests are converted into agricultural land, as this harms biodiversity [54]. Land conversion can also harm regulatory services that forests normally offer to local communities due to their water retention properties. Extreme weather events such as heat, floods or drought can have direct impacts on mortality, but these events can also affect biodiversity and ecosystem services such as food and fuel provision, affecting health and wellbeing [66]. Climate change is likely to promote an increase in food prices, by extreme weather events damaging crops and this will disproportionally affect lower income groups as (healthy) foods become scarce and more expensive [67]. 

#### 3.2.5. Inequalities and Context

The INHERIT model emphasizes inequalities between individuals and groups by including icons with individuals standing at different levels throughout both pathways, and by including health inequalities as a resulting impact of the pathways. The mix of interacting drivers at the beginning of the pathways contribute to inequalities, because this driver mix impacts differently on locations and populations, creating physical environments that vary between places and populations in society. In addition, the pathways are influenced by demographic, social and economic contexts [8]. This context (visualized as a grey area surrounding the model) contributes to the large variation in the way individuals and groups are exposed to environmental factors and the way they experience them. The different exposures and experiences lead to different health and wellbeing impacts between individuals and groups. For example, socio-economic aspects such as income and education have been found to modify whether, and to what extent, a person or population group is exposed to a physical environment, and the extent to which this exposure leads to a certain health and wellbeing impacts [13,68,69]. In addition groups, including children, the elderly, and people with existing health conditions may be more susceptible to health damaging impacts of environmental exposures.

#### 3.2.6. Food Example of Inequalities

Applying the aspect of inequalities to the case of food consumption, certain drivers and resulting pressures lead to greater access to unhealthier food choices in lower-income communities. For example, the higher number of fast food outlets in deprived neighborhoods and the higher costs of healthier foods than unhealthy alternatives (with fruits and vegetables being up to 40% more expensive in poor neighborhoods) [70,71,72]. Thus, these lower-income communities are exposed to less healthy environments, and together with other factors, this may cause lower-income groups to have less healthy diets. This is supported by evidence suggesting it is increased energy intakes, and not decreased physical activity that drives the current obesity epidemic among lower socioeconomic groups [72]. Diseases associated with obesity explain much of the premature mortality and loss of healthy years lived among lower socioeconomic groups. What is more, the gap between the rich and poor in terms of obesity rates is increasing, resulting in more health inequalities [72].

#### 3.2.7. Feedback Loop

One of the challenges of modelling any system, including simple chains of cause and effect, is how to represent the reality that outputs of the system will feedback to become inputs to the same system. In the INHERIT model, a single feedback loop is included to show that key system outputs —changes in effects on health, wellbeing and inequalities—may lead back to feature amongst the multiple interacting factors (inputs) which drive the system. For example, improved health status within the population may lead to accelerated economic productivity, which may increase pressures on the environment and ecosystems. While it is recognized that other feedback loops can be conceived, it has been resolved to omit these from INHERIT to preserve the simplicity and utility of the model.

#### 3.2.8. Behavior in the INHERIT Model

It is crucial to identify and understand the behaviors that have an impact on both health and the environment. Furthermore, it is important to understand the system and context in which these behaviors occur, and how they differ between individuals and population groups, as it can help to explain health inequalities. These insights can help in identifying (inclusive) solutions that do not have unintended consequences on different fields. In the INHERIT model, two behavioral components can be identified: behavioral hotspots (depicted as magnifying glasses at three points in the model) and the Behavioural Change Wheel (on the left side of the model). In the following sections, these behavioral components will be further explained.

#### 3.2.9. Behavioral Hotspots and Food Examples

Regarding the hotspots, the first one is among the drivers, as drivers are related to and interact with behaviors and lifestyles. It is an important entry point for action as the pathways are initiated here. Behaviors and lifestyles are one of the drivers of current food systems and they interact with other drivers. For example, in most Western industrialized societies, eating large amounts of meat is a widespread, valued practice for consumers, and as living standards increase globally, the demand for meat increases [73]. Also, there is a trend to eat out more often, with less time spent on purchasing and cooking food at home [74,75]. This interacts with the food service industry, stimulating the development of more food outlets. Consumers in high-income countries now expect, value and demand the availability of cheap, varied food throughout the year [54]. These drivers interact with other drivers, such as increasingly pervasive food marketing, the food industry developing more processed foods, powerful retail industries and food pricing, which influence and are influenced by consumer demand [2,52].

Besides the behavioral hotspot among the drivers, there are two additional behavioral hotspots in the lower part of the pathway (indicated by magnifying glass icons) to make explicit that, at these points behavior modifies the relationship between the physical environment and effects on health and wellbeing, besides other contextual modifiers. The second hotspot influences whether and to what extent one is exposed to an environment: living in a community with a high number of fast food outlets and restaurants, does not automatically mean an individual will become overweight or develop related non-communicable diseases. One must actually interact with this environment, such as actually going into a fast food restaurant for dinner instead of doing shopping for groceries and cooking and eating at home.

The third hotspot influences the extent to which this exposure leads to health and wellbeing impacts: if one eats out often, this again does not mean someone will inevitably experience negative health impacts. Compensatory behaviors also play an important role, such as whether someone makes healthy choices when eating out or compensates caloric intake during the other meals of the day or if one increases physical activity on that day. 

In the long term, differences in these behaviors (and the environments that influence behavior) between individuals and population groups contribute to health inequalities. For example, one’s level of education has a strong influence on household food choices, purchases and eating patterns [76]. Low-income families are more likely to buy cheaper and more energy-dense, processed foods, which are often poor in nutrients. They are also less likely to buy fruits and vegetables regularly [76,77]. Long working hours and low income may pressure disadvantaged families to engage in these purchase behaviors. 

#### 3.2.10. Behavioural Change Wheel (BCW)

**Figure 2:**
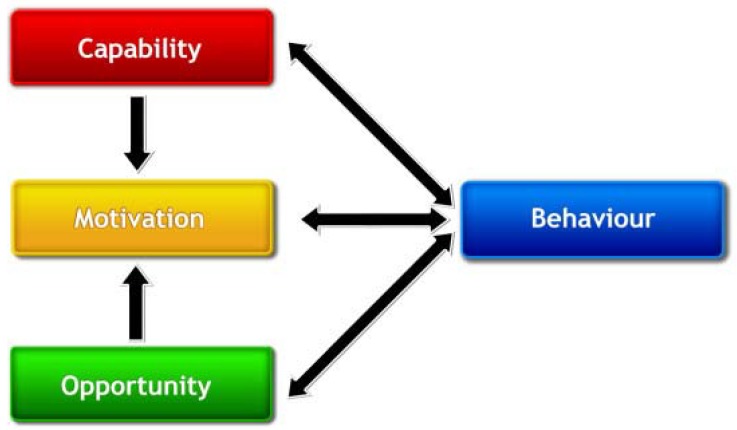
The COM-B system, source: [49].

The Behavioural Change Wheel on the left side of the model starts with the question: “What conditions internal to individuals and their social and physical environments need to be in place in order for a specific behavioral target to be achieved?” The behavioral part, which lies in the core of the BCW, is represented by the COM-B system, which stands for capability, opportunity, motivation and behavior (see Figure 2 for the COM-B system) [47]. Traditionally applied to health-related behaviors, this system is considered sufficiently broad to be applied to behavior in any setting [47] and a recent paper showed how the COM-B model may be applied in the case of environmental related behavior (e.g., recycling) [78]. The COM-B system can be applied to any level: individuals, groups and populations, and not only target populations but decision makers as well. See Table 2 for an explanation of the COM-B system using the example of food consumption. In order to understand behavior, it is essential to perform an analysis of these three conditions and to look for differences in these three conditions between individuals and population groups. It has been estimated that a substantial share of variation in life expectancy between the least and most materially and socially deprived in high-income countries can be explained by social patterning of health-related behavior [79]. Besides differences between individuals and population groups in aspects such as the capability to perform certain behavior, physical and social environments provide different opportunities to perform certain behavior [12,68]. The BCW can help analyze these differences in behavior to better understand (health) inequalities between individuals and populations.

The INHERIT model can also be used to guide identification and development of effective interventions and policies, using the BCW outer rings. When the specific behavioral elements that need to be targeted have been identified, the most effective interventions functions to change the elements of relevance can be selected from the ring surrounding the COM-B. This ring contains nine intervention functions, which are ways in which an intervention can change behavior. The policy categories in the outer ring support and enable these intervention functions. Intervention functions can be used for multiple behavioral elements, and a combination of intervention functions can be used to target one behavioral element. For an explanation of all nine functions and the seven policy categories and how they link to the behavioral elements, see Michie et al. [47,49].

Applying the BCW to the example of food consumption, a behavior to be changed could be eating less red and processed meats and more plant-based meals. Analysis of COM-B elements may show that meat eating is largely habitual and influenced by social norms, and habits are greatly influenced by environmental cues and contexts [81]. This means interventions functions should be chosen that target automatic motivation and social and physical opportunity [47]. An intervention function to address physical opportunity is incentivisation (creating an expectation of reward), and a policy category to enable this intervention is fiscal measures. In this case, a taxation of red and processed meat could be implemented. However, to avoid widening (health) inequalities, it should be investigated whether behavioral differences follow a social gradient. For example, taxes could affect those with lower incomes disproportionately, as they already spent a greater share of their income on food, which could increase health inequalities. Combining taxes on unhealthy and unsustainable foods such as red meats, with subsidies on healthy and sustainable foods such as plant-based meat substitutes, could reduce the risks of widening health inequalities [72]. This combination could also compensate for low acceptance of taxation measures [85].

### 3.3. Applying the INHERIT Model

The INHERIT model has been applied to a case study on the implementation of sustainable food in nursery schools in the city of Madrid (Spain), where ecological food products will be introduced in 56 nursery schools and cantinas will adapt their menus to offer healthier and more sustainable food (including locally produced food). This project has been implemented in the context of the Milan Food Policy Pact [86] and is one of the pilot projects that will be evaluated for the INHERIT project. The case study illustrates how the INHERIT model can be used to map the impacts of actions using the different elements of the model. 

An analysis of the physical environment of this case study showed that urban areas are prone to obesogenic environments caused by diverse socioeconomic and demographic drivers [87]. Controlled environments such as nursing schools could provide an opportunity for disconnection from such pressures. Nevertheless, this potential was not exploited before, and less healthy and sustainable food patterns have been transferred into these environments [88]. Multidimensional approaches have higher opportunities for success due to diversity in targets. The actions in this case study offer an example of various policy categories (regulation, service provision, guidelines and communication) and intervention functions (training, enablement, education and incentivization) from the BCW. 

In order to determine how actions affect health and wellbeing, the INHERIT model has been applied to identify several mechanisms and their interactions, through both proximal and distal pathways. In this case, normative action would have a direct impact on wellbeing through changes in food availability (e.g., achieving a proper caloric intake, a higher proportion of nutrients per meal and lower meat-based protein intakes). Such changes have an impact on the proximal environment of the target population by increasing acceptance of several food items, increasing both motivation and opportunity to eat these food items [89,90]. However, this is not enough to change the food environment, as education and training is required in order to translate those changes into wider environments such as households, particularly in deprived areas where habits (automatic motivation) in terms of food consumption may be more difficult to change [91]. At the distal level, actions such as diminishing intermediaries or the reduction of animal-derived proteins may reduce environmental impacts derived from food consumption. These measures would have impacts on ecosystems due to potential decreases in CO_2_ emissions derived from food transport and GHG emissions from the agricultural sector [92,93]. It could also have an impact on the trend towards agricultural land abandoning. In addition, reducing intraurban health inequalities, particularly at early ages, may have positive impacts on for example demographic and social structures (e.g., integration of neighborhoods) and the economy (e.g., lower work and academic absenteeism).

### 3.4. Results from Expert and Stakeholder Consultations

Feedback from two consultation sessions upon presenting the INHERIT model was generally positive, with experts perceiving the model as a useful tool to think about links between environment, health and equity problems. It was considered helpful in identifying entry-points for actions and in identifying impacts and interrelationships. Key findings from the workshops included that it was not immediately clear whose behavior the model should be applied to. This is not made explicit in the model itself, as it is both about behavior of target populations and decision-makers. Another point made was that the model should make clear that intersectoral cooperation, and bringing people from health and environment together, is an important part of action. One of the evaluation workshop outcomes was that the model, viewed in isolation, was too complex to understand on its own, as it is very broad and all compassing. A recommendation from the workshops was to make a short guide to explain the model.

## 4. Discussion

Many aspects of contemporary human behaviors and lifestyles and the driving forces that shape them are generally both unhealthy and damaging to the environment. The environment in turn affects our health and wellbeing, either directly or by influencing our behavior. Moreover, environments and behaviors differ between individuals and populations, contributing to health inequalities. For the INHERIT project, a conceptual model was developed to help navigate the complex interrelationships between health, sustainability, equality and behavior. The INHERIT model integrates insights from the ecological public health domain and the behavioral domain to analyze and understand complex interrelationships, and to find inclusive solutions to tackle the issues stemming from these complex interrelationships. In developing the model, we have employed examples from food production and food consumption patterns to demonstrate how the model can help identify and clarify the complex interrelationships between current behaviors and lifestyles, health and environmental impacts and inequalities. In addition, the model can help identify potentially effective actions in the area of food consumption, including possible unintended consequences for different fields that risk being overlooked. However, we believe the model can be applied to a wide range of fields that involve health, environmental sustainability and equity and in which behavior is thought to play an important role, such as active transport and physical activity, energy efficient housing and green space [92,93,94,95,96]. 

### 4.1. Integration of Fields

An advantage and innovative aspect of the INHERIT model is the combination and integration of two models used in different domains: an established behavioral change model was combined with an ecological public health model. Human behavior and its determinants are not always taken into account when dealing with environmental health and sustainability problems, despite behavior’s pivotal role in these problems. Others have also argued for the need for a framework that more explicitly incorporates modern lifestyles as a common cause and bridge between public health and environmental perspectives [4]. The INHERIT model explicitly includes behavioral features to encourage taking the role of behaviors into account when analyzing (solutions for) health and sustainability issues. In addition, an inequality aspect was added to encourage thinking about the higher-level drivers that lead to inequalities and to promote inclusive thinking when designing interventions and policies to avoid widening the gap between social groups. This integration of fields stimulates thinking outside one’s own field, enabling new insights to be gained on interrelationships and possible solutions, while avoiding unintended consequences for other fields than one’s own. Recently, an INHERIT policy roadmap was published by colleagues [97], for which the INHERIT model was used as a guidance for further policy actions. Feedback from an expert workshop on this roadmap was that the attention drawn to distal impacts and behavior was found very valuable in selecting appropriate policy actions (both upstream, downstream, both structural and psychological). 

The INHERIT model can help understand the linkages and actions contributing to the Sustainable Development Goals (SDGs), the 17 global goals set by the United Nations in the 2030 Agenda, targeting people, planet and prosperity [98]. For example, when considering solutions to solve health problems from eating red meat, one may turn to fish consumption as it has many health benefits. However, one must consider environmental impacts as well. Overfishing can result in over-exploitation of marine ecosystem services, which makes it important to focus on sustainable fishery options such as aquaculture and working with ecolabels or certification schemes [99,100]. By thinking about behavior and equity, one may identify differences in opportunities such as affordability (sustainable fish can be too expensive for low socioeconomic groups) and accessibility (in more deprived communities, sustainable fish may not be available), but also differences in for example knowledge or cultural acceptability. 

### 4.2. Behavior and Inequalities

As changing behaviors and lifestyles is an essential part of the transition to healthier, more sustainable societies, the action focus of the BCW makes it particularly useful. The BCW, combined with the behavioral hotspots in the model, can guide systematic analysis of behaviors involved in health and sustainability issues, and the BCW promotes an awareness of all potential intervention functions and policy categories [47]. The inequality component of the INHERIT model adds an awareness of inequalities, guiding identification of inclusive action and avoiding unintended consequences that may widen the inequality gap. In the environmental health field, often solely differences in exposures to environmental risks are described, while insights into the mechanisms behind these differences are required in order to tackle inequalities effectively. As described by Kruize et al. [12], environmental health inequalities are created at different levels, from macro or societal levels (e.g., socioeconomic stratification and residential segregation) to community and neighborhood level (e.g., differences in physical and psychosocial risks and exposures) to individual level (e.g., differences in vulnerability and coping strategies). It is essential to recognize the role of structural (social, economic, political) drivers in producing environmental and health inequalities, but also of health related behaviors and lifestyles as mediators between the environment and health inequalities [12]. The INHERIT model reinforces the importance of an ecological/systems perspective in which health inequalities do not result only from individual (including behavioral) factors, but also from factors outside the individual, at different levels ranging from interpersonal, societal to supranational. It argues for consideration of the influences of higher-level systems and contexts in which health inequalities exist [12,18,69,101]. Therefore, this model is not about giving sole responsibility to unhealthy, unsustainable behaviors of the individual. On the contrary, it is also about the system level drivers and environments that create and maintain these behaviors, and about the responsibility of decision makers and policymakers who influence these drivers and environments. The COM-B model can also be applied to and used in discussion with decision-makers and implementers, as for them to take action, they too must be motivated, capable and have social and physical environments that give them an opportunity to take action. 

### 4.3. Bringing Together People from Different Sectors

Although the complex nature of health and environmental issues requires multi-sectoral action, the health and environmental (but also urban planning, transport, food chain, education) sectors often still work in silos, including the research fields of the sectors [24], which results in fragmentary knowledge, interventions and policies. Therefore, conceptual models can be important tools to bring together fragmentary knowledge, promoting an integrated understanding of, and directing action towards, complex issues. Conceptual models can be useful tools to think with [30] and when applied to specific issues, the family of DPSEEA models have been shown to be effective in engaging multidisciplinary stakeholders [31,32]. Conceptual models can also support intersectoral cooperation by facilitating a common understanding and language, promoting dialogue between disciplines and adoption of a shared recognition of roles and responsibilities. The INHERIT model can support the Health in All Policies, Environment in All Policies and Whole-of-Society approaches, as a tool to enhance understanding [25,26,27]. It can provide a certain structure that can be used to gain insights and unravel the complexity of current health and sustainability issues. In addition, the model can potentially facilitate dialogue and debates, define problems and develop multidisciplinary solutions.

### 4.4. Complexity and Limitations of the Model

One of the stakeholder consultation outcomes was that some stakeholders found that the model is a little too complex to understand on its own, as it is very broad and all encompassing. Rutter et al. [102] recently alluded to the challenge of complexity in public health and the need for innovative solutions when they observed, “The identification, implementation and evaluation of effective responses to major public health challenges require a wider set of approaches and a focus on complex systems”. The discussion of conceptual models in this paper takes place against this backdrop of inherent complexity and an acceptance that, despite the rhetoric, systems research in public health is in its infancy [103]. The INHERIT model undoubtedly retains strong elements of linearity in an effort to preserve the utility which comes with simplicity. For example, some arrows in the model are unidirectional (e.g., from pressures on physical environments to pressures on ecosystems), whereas in reality, bidirectional relationships may exist between elements of the model. Moreover, only one feedback loop is visualized in the model (from impacts back to drivers), while in reality, several feedback loops may exist depending on the specific issue one analyzes. By omitting these loops, the model increases in simplicity and utility, but like any other model, it is not a precise, true representation of reality. It is a tool to think with, and a tool that facilitates understanding of the interrelationships in complex intersectoral challenges and opportunities involving health, sustainability, inequality and behavior. As working intersectorally has proven to be a challenging undertaking in itself, providing models that are simple and high in utility seems particularly important to stimulate intersectoral cooperation, research and policymaking. A conclusion from the expert consultations was that while omission of the feedback loops enhances the accessibility of the model, supporting material contains a recognition of the potential for such loops to exist. However, the model is configured to take account of the complexity in the determinants of health, wellbeing, equality and sustainability. A recommendation from the workshops was to make a short guide to explain the model. To make the model easier to understand, a user’s manual was written, which is to be published online, together with an online webpage and animated version of the model that include short explanations of the model for use in stakeholder discussions. A promising addition may be the development of a simple (online) training course in working with the model. In addition, this paper can serve to explain the model for those interested in using it. An additional limitation of the model is that, because it is an integration of two models, it is likely to be used only by people with an interest in combining research in the field of public health and environment. However, it is important that independent of the disciplinary background of the user, these separate issues are studied in an integral manner. Promoting awareness about the importance of integral thinking and action among any researcher or policy-maker practicing public or environmental health is therefore essential. Integral models such as the INHERIT model can be used as tools to support such promotion.

The INHERIT model is a relational framework which shows how the different study domains are interrelated and how these fields of studies should be approached as such. It is not intended as a tool to couple methods and data from different fields with one another, but individual fields of study can bring their own methods and data to the table and deploy these to address the multifaceted societal problems that confront us in the Anthropocene. In essence, the utility of the INHERIT model is founded on its capacity (through promoting a shared conceptualization of the issues and identifying entry points for action) to enable society to exploit very different datasets and discipline-specific methodologies and raise awareness for interdisciplinary cooperation. It supports the integrated “whole of society” collaborations for health, wellbeing and equity that are necessary.

### 4.5. Future Directions

Future researchers and policy-makers are already increasingly using a multidisciplinary approach, with governments taking shared responsibilities, for example the Dutch Food Agenda (Voedselagenda) where multiple governmental departments are working together with industry, producers, education, and more [104]. Pursuing an integral approach with special attention for inequalities and behavior, will be a key activity to understand and develop efficient, effective actions for current and future challenges of health and sustainability, while preventing unintended consequences. Integral models such as the INHERIT model can support this pursuit, by offering entry points for integral action and promoting a shared view of health and sustainability challenges. The approach proposed in this paper may find practical application in many areas, and it is certainly intended to understand how human behavior change offers a potential solution to many challenges. 

The INHERIT model has been and will be further tested in the INHERIT project: it has been used to identify triple win practices throughout Europe (http://inherit.eu/resources/about-database/), has guided the INHERIT policy road map for effective triple win policy interventions [97] and it will be used to implement and evaluate triple win pilots throughout Europe, including qualitative evaluations of intersectoral cooperation. Moreover, the activities carried out throughout the project will provide additional insights into the utility of the model as a tool to identify and develop effective triple win policies and action. These activities will test the theoretical and practical applicability of the model regarding different challenges such as food and energy consumption, green space or active travel. In addition, the model will be included in a policy toolkit for policy makers, which will further promote its uptake in the policy context. While the current configuration and components of the model are likely to remain unchanged, opportunities will be taken to introduce refinements to the model. 

## 5. Conclusions

Currently, behavior, health equity and environmental sustainability are often not considered in a coherent way when developing policies or interventions. This has the potential to create tensions and conflicting outcomes. The INHERIT model is unique as it brings together these different but interrelated worlds and can help to clarify interrelationships and identify effective actions aimed at behaviors and lifestyles. It promotes the identification of relationships that might otherwise be overlooked. Thereby, it can contribute to and clarify the highly multidisciplinary SDGs as set in the 2030 Agenda. The INHERIT model is a significant step towards engaging in a more interdisciplinary approach to understanding and finding solutions for current complex societal challenges. This will create new opportunities to improve health and health equality, while ensuring environmental sustainability, thus attaining a triple win.

## Figures and Tables

**Table 1:** Differences between DPSEEA models and INHERIT model.

Model	Key Contribution	Critical References
DPSEEA	Presents simple linear environmental health pathway from drivers−pressures−state−exposures−effects to action	[35,40]
Modified DPSEEA (mDPSEEA)	Explicitly considers the role of context in modifying exposure risk	[8]
Ecosystems-enriched DPSEEA (eDPSEEA)	Extends temporal and spatial scope by considering impacts of threats to health in the here and now (proximal pathway), as well as impacts on health of future generations or populations far away from environmental changes associated with accelerating damage to planetary processes and systems (distal pathway)	[34]
INHERIT model	Builds on mDPSEEA and eDPSEEA by explicitly considering behavioral change and (health) equalities	Current paper

**Table 2:** Explanations of the COM-B elements illustrated by examples from food consumption.

COM Element	Explanation and Illustration by Examples from Food Consumption
Capability	Being psychologically and physically able to perform a behavior, such as having the necessary knowledge of what a healthy, sustainable diet entails and having a set of skills that allows cooking healthy, sustainable meals. Differences in economic resources may explain differences in dietary choices between socio-economic status groups.
Opportunity	Having a physical and social environment that makes it affordable, acceptable, and accessible to perform a behavior. For example, parents encouraging their children to eat fruit on a daily basis, or growing up in a family where eating meat only twice a week is considered normal. Living in an area with a market that has a wide variety of affordable fruit and vegetables can increase consumption, which may explain some of the differences between socioeconomic groups regarding consumption of fruit and vegetables [80].
Motivation	All brain processes that make someone motivated to select behavior (over other behaviors) at the relevant time. It includes reflective processes, such as consciously deciding to eat vegetarian meals twice a week, or having certain attitudes towards eating meat. It also includes automatic processes, involving more impulsive, emotional processes and habits. For example, a large part of dietary behaviors is habitual, performed without much conscious thought [81]. To influence actual behavior, motivation has to be strong at the right time, so behavioral intentions do not always actually lead to performing the behavior. People have competing wants and needs and stronger non-environmental motivations can be barriers to pro-environmental or healthy behavior. Think of the intention to eat healthy salads for lunch all week, but falling for the hamburger that smells so good in the canteen [82,83]. Socio-economically disadvantaged individuals appear more motivated by price and familiarity, and less by health concerns, compared to higher income individuals [84].
Interaction between the COM elements	The three elements interact: capability and opportunity can influence motivation. For example, an environmental cue at the right time when purchasing meat (e.g., a large variety of meat-substitutes in the same supermarket aisle) can increase motivation to buy a meat-substitute through increased (physical) opportunity. Subsequently, being more motivated to perform a certain behavior may lead to doing things that increases the capability to do so (e.g., wanting to learn how to prepare the meat-substitutes leads to practicing cooking skills). Behavior can also influence the COM elements, for example when someone is cooking and considers it a pleasant thing to do, the motivation to continue cooking may increase.
